# Supplementary material for: Extracellular Vesicles Derived from Bone Marrow in an Early Stage of Ionizing Radiation Damage Are Able to Induce Bystander Responses in the Bone Marrow
Source: Cells. 2022 Jan 4;11(1):155. doi: 10.3390/cells11010155 (PMC8750882; doi:10.3390/cells11010155)
Supplement: Supplementary file 1 [file cells-11-00155-s001.zip › cells-1520514 resubmitted Supplementary.pdf]

Supplementary Materials:

Supplementary Table S1: Pathways targeted by significantly altered miRNAs, isolated from BM-derived EVs isolated 24h after irradiation. miRNAs were associated with their target, using Diana mirPath (microT-CDS). Pathways with a p-value < 0.05 and MicroT threshold < 0.8 were calculated. KEGG pathway identifiers are included in parenthesis.

KEGG pathway	p-value	genes	Number of miRNAs	miRNAs regulating the pathways
Hippo signaling pathway (mmu04390)	3.55E-08	30	6	mmu-miR-33-3p, mmu-miR-375-3p, mmu-miR-140-3p, mmu-miR-152-3p, mmu-miR-199a-5p, mmu-miR-669o-5p
FoxO signaling pathway (mmu04068)	1.66E-05	27	6	mmu-miR-33-3p, mmu-miR-375-3p, mmu-miR-140-3p, mmu-miR-152-3p, mmu-miR-199a-5p, mmu-miR-669o-5p
Proteoglycans in cancer (mmu05205)	1.66E-05	30	6	mmu-miR-33-3p, mmu-miR-375-3p, mmu-miR-140-3p, mmu-miR-152-3p, mmu-miR-199a-5p, mmu-miR-200c-5p
Signaling pathways regulating pluripotency of stem cells (mmu04550)	8.56E-05	22	6	mmu-miR-33-3p, mmu-miR-375-3p, mmu-miR-140-3p, mmu-miR-152-3p, mmu-miR-199a-5p, mmu-miR-669o-5p
PI3K-Akt signaling pathway (mmu04151)	3.83E-04	44	6	mmu-miR-33-3p, mmu-miR-375-3p, mmu-miR-140-3p, mmu-miR-152-3p, mmu-miR-199a-5p, mmu-miR-669o-5p
Wnt signaling pathway (mmu04310)	4.46E-04	19	7	mmu-miR-33-3p, mmu-miR-375-3p, mmu-miR-140-3p, mmu-miR-152-3p, mmu-miR-199a-5p, mmu-miR-669o-5p, mmu-miR-200c-5p
AMPK signaling pathway (mmu04152)	1.06E-03	20	6	mmu-miR-33-3p, mmu-miR-375-3p, mmu-miR-140-3p, mmu-miR-152-3p, mmu-miR-199a-5p, mmu-miR-669o-5p
Hypertrophic cardiomyopathy (HCM) (mmu05410)	1.96E-03	17	6	mmu-miR-33-3p, mmu-miR-375-3p, mmu-miR-140-3p, mmu-miR-152-3p, mmu-miR-199a-5p, mmu-miR-669o-5p
Melanogenesis (mmu04916)	2.88E-03	17	7	mmu-miR-33-3p, mmu-miR-375-3p, mmu-miR-140-3p, mmu-miR-152-3p, mmu-miR-199a-5p, mmu-miR-669o-5p, mmu-miR-200c-5p
Insulin signaling pathway (mmu04910)	5.19E-03	22	7	mmu-miR-33-3p, mmu-miR-375-3p, mmu-miR-140-3p, mmu-miR-152-3p, mmu-miR-199a-5p, mmu-miR-669o-5p, mmu-miR-200c-5p
Neurotrophin signaling pathway (mmu04722)	9.07E-03	20	6	mmu-miR-33-3p, mmu-miR-375-3p, mmu-miR-140-3p, mmu-miR-152-3p, mmu-miR-199a-5p, mmu-miR-669o-5p

Dilated cardiomyopathy (mmu05414)	9.07E-03	16	7	mmu-miR-33-3p, mmu-miR-375-3p, mmu-miR-140-3p, mmu-miR-152-3p, mmu-miR-199a-5p, mmu-miR-669o-5p, mmu-miR-200c-5p
MAPK signaling pathway (mmu04010)	1.23E-02	32	7	mmu-miR-33-3p, mmu-miR-375-3p, mmu-miR-140-3p, mmu-miR-152-3p, mmu-miR-199a-5p, mmu-miR-669o-5p, mmu-miR-200c-5p
Adrenergic signaling in cardiomyocytes (mmu04261)	1.24E-02	19	7	mmu-miR-33-3p, mmu-miR-375-3p, mmu-miR-140-3p, mmu-miR-152-3p, mmu-miR-199a-5p, mmu-miR-669o-5p, mmu-miR-200c-5p
HTLV-I infection (mmu05166)	1.45E-02	30	7	mmu-miR-33-3p, mmu-miR-375-3p, mmu-miR-140-3p, mmu-miR-152-3p, mmu-miR-199a-5p, mmu-miR-669o-5p, mmu-miR-200c-5p
Pantothenate and CoA biosynthesis (mmu00770)	2.20E-02	3	2	mmu-miR-33-3p, mmu-miR-140-3p
Hedgehog signaling pathway (mmu04340)	2.20E-02	10	5	mmu-miR-33-3p, mmu-miR-140-3p, mmu-miR-152-3p, mmu-miR-199a-5p, mmu-miR-200c-5p
TGF-beta signaling pathway (mmu04350)	2.20E-02	13	6	mmu-miR-33-3p, mmu-miR-375-3p, mmu-miR-140-3p, mmu-miR-152-3p, mmu-miR-199a-5p, mmu-miR-669o-5p
Renal cell carcinoma (mmu05211)	3.61E-02	11	5	mmu-miR-33-3p, mmu-miR-375-3p, mmu-miR-140-3p, mmu-miR-152-3p, mmu-miR-199a-5p,
Protein processing in endoplasmic reticulum (mmu04141)	3.67E-02	20	6	mmu-miR-33-3p, mmu-miR-375-3p, mmu-miR-140-3p, mmu-miR-152-3p, mmu-miR-199a-5p, mmu-miR-669o-5p
Pathways in cancer (mmu05200)	3.67E-02	40	7	mmu-miR-33-3p, mmu-miR-375-3p, mmu-miR-140-3p, mmu-miR-152-3p, mmu-miR-199a-5p, mmu-miR-669o-5p, mmu-miR-200c-5p
Glycosphingolipid biosynthesis - lacto and neolacto series (mmu00601)	3.86E-02	3	3	mmu-miR-33-3p, mmu-miR-152-3p, mmu-miR-199a-5p
Basal cell carcinoma (mmu05217)	4.43E-02	10	5	mmu-miR-33-3p, mmu-miR-375-3p, mmu-miR-140-3p, mmu-miR-152-3p, mmu-miR-199a-5p
D-Arginine and D-ornithine metabolism (mmu00472)	4.53E-02	1	2	mmu-miR-199a-5p, mmu-miR-669o-5p
